# Prescription opioid injection among young people who inject drugs in New York City: a mixed-methods description and associations with hepatitis C virus infection and overdose

**DOI:** 10.1186/s12954-020-00367-2

**Published:** 2020-03-30

**Authors:** Pedro Mateu-Gelabert, Honoria Guarino, Jon E. Zibbell, Jennifer Teubl, Chunki Fong, Elizabeth Goodbody, Brian Edlin, Carli Salvati, Samuel R. Friedman

**Affiliations:** 1 CUNY Graduate School of Public Health and Health Policy, Institute for Implementation Science in Population Health (ISPH), 55 West 125th Street, New York, NY 10027 USA; 2 RTI International, 2987 Clairmont Road, Century Plaza 1, Suite 400, Atlanta, GA 30329-4434 USA; 3 National Development Research Institutes, Inc., 71 West 23rd St, New York, NY 10010 USA; 4 Independent Consultant, New York, NY USA; 5 NYU School of Medicine, 550 First Avenue, New York, NY 10016 USA

**Keywords:** Prescription opioid misuse, Prescription opioid injection, Young PWID, Heroin, Drug overdose, Hepatitis C virus (HCV) infection

## Abstract

**Aim:**

Evidence is emerging that prescription opioid (PO) injection is associated with increased health risks. This mixed-methods study compares the mechanics of PO and heroin injection and examines the demographic and drug-related correlates of lifetime PO injection in a sample of young people who inject drugs (PWID) in New York City (NYC).

**Methods:**

Qualitative analysis of 46 semi-structured interviews with young adult opioid users ages 18–32. Interview segments describing PO injection were analyzed for common themes. Quantitative analysis of structured interviews with 539 young adult opioid users ages 18–29 recruited via respondent-driven sampling (RDS). Analyses are based on the subsample of 353 participants (65%) who reported having ever injected drugs. All variables were assessed via self-report, except hepatitis C virus status, which was established via rapid antibody testing.

**Results:**

Participants described injecting POs and reported that preparing abuse-deterrent pills for injection is especially cumbersome, requiring extended manipulation and large amounts of water. Injecting POs, in contrast to injecting heroin, requires repeated injections per injection episode. Among RDS-recruited participants, the majority of injectors reported injecting POs, sporadically (33%) or regularly (26%), but often infrequently (≤ 7 days/month). In separate multivariable analyses controlling for syringe- and cooker-sharing, ever injecting POs was a significant predictor of testing HCV antibody-positive (AOR = 2.97) and lifetime experience of non-fatal overdose (AOR = 2.51). Ever injecting POs was independently associated with lifetime homelessness (AOR = 2.93) and having grown up in a middle-income ($51,000–100,000/year vs. ≤ $50,000/year; AOR = 1.86) or a high-income household (> $100,000/year vs. ≤ $50,000/year; AOR = 2.54).

**Conclusions:**

Even in an urban environment like NYC with widespread heroin access, most young PWID have injected POs, although less frequently than heroin. PO injection involves practices that are known to increase risk for blood-borne viral infection (e.g., repeated injections) and predicted testing HCV-positive, as well as overdose. PO injection may also serve as a marker for a subgroup of PWID at elevated risk for multiple drug use-related comorbidities. Programs that provide prevention services to PWID need to tailor harm reduction measures and messaging to the specific practices and harms associated with the injection of POs.

## Introduction

Prescription opioid (PO) injection is on the rise in the USA. Among a nationally representative sample of people 12 years or older, the prevalence of lifetime PO injection increased from 1.6 per 1000 in 2003–2005 to 2.7 per 1000 in 2012–2014 [1]. This trend is also reflected in US PO-related treatment admissions, with PO injection among new admissions to substance use treatment rising from 11.7% in 2004 to 18.1% in 2013 [2].

The prevalence of PO injection among non-treatment-seeking samples of drug users varies across regions of the USA. In rural Kentucky, 89% of people who inject drugs (PWID) in a cohort sample reported injecting POs in their lifetime [3], while a study of young, street-involved PWID in urban New York City (NYC) and Los Angeles who reported recent nonmedical PO use found that 72% reported having ever injected POs (“lifetime” PO injection) [4]. In Scott County, Indiana, the site of a large HIV outbreak among PWID in 2015, the vast majority of HIV-infected PWID reported the current injection of extended-release oxymorphone (OpanaER®) as their primary drug of choice [5]. In contrast, a study of young PWID in rural New York State who misused POs [6] reported lower rates of current PO injection (58%). Differences in study design and PO availability across regions are likely influencing the reported variability in PO injection rates.

Beyond the risks associated with injection drug use in general, which include exposure to blood-borne viruses (such as HIV and hepatitis C virus [HCV]), as well as bacterial infections related to injecting in unsterile environments [7], the injection of PO pills in particular has been shown to be independently associated with HCV infection in samples of PWID [6, 7]. Several recent studies have found that PWID who report injecting POs have an increased likelihood of being HCV antibody-positive relative to PWID who inject heroin only [3, 4, 6, 8, 9]. A prospective study of drug-using street youth, however, found in a multivariate model that PO injection was not a significant predictor of HCV incidence [10].

While injection-related viral transmission risk has largely been associated with the sharing of contaminated injection equipment (e.g., syringes and cookers), the risks associated with PO injection may occur at each stage of the process involved in preparing and injecting opioid pills, including pill crushing, the use of extra water (relative to heroin injection), sharing “rinse shots,” and other practices that place pill injectors at increased vulnerability to viral exposure [8]. Most recently, the cumbersome and lengthy process of preparing and injecting POs has been shown to often involve multiple injections per injection episode which may contribute to the development of new, blood-borne viral transmission pathways [11, 12].

In a systematic review of health outcomes associated with PO injection, Lake and Kennedy [9] report that PO injection has also been found to be correlated with recent non-fatal overdose among women (not men) in a cohort study of PWID recruited through street outreach [13] and to have marginally significant associations with lifetime non-fatal overdose among street-recruited rural drug users [14]. One study found that PO injection among street-recruited youth who used POs was correlated with lifetime overdose in bivariate analyses, yet did not remain significant in multivariable analysis [15], while other studies among street-recruited [16, 17] and syringe exchange program-recruited opioid users [18] did not find any significant association between PO injection and recent non-fatal overdose.

While prior studies have described the mechanics of PO injection and have found significant associations between PO injection and HCV-positive status or overdose, in this paper, we expand on this literature by using a mixed-methods design that allows for a more comprehensive understanding of PO injection. Specifically, we employ qualitative interview data describing the mechanics of preparing and injecting POs to help interpret and contextualize our quantitative findings addressing the research question: “What are the associations between PO injection and HCV infection and unintentional opioid overdose?” We also present additional quantitative data to compare patterns of heroin injection vs. PO injection in the context of young people’s opioid use trajectories. Furthermore, our distinctive study samples of sociodemographically diverse, urban young adults who were actively using opioids, but were not necessarily street-involved, help fill an important gap in our understanding of the opioid injection patterns of young opioid consumers living in American cities.

## Methods

This mixed-methods paper presents select findings from a larger study that assessed the drug use practices and health risks of young adults in NYC who use opioids (including the nonmedical use of POs and/or heroin use). The current analyses focus on patterns and correlates of PO injection in two samples of young adults who use opioids. Qualitative data from one sample are presented to characterize the mechanics used to prepare and inject POs. Quotations from face-to-face interviews with young people who inject POs and heroin are presented to identify behaviors and techniques specific to the way PO pills are prepared and injected in order to differentiate them from the techniques used with heroin and other illicit drugs. Quantitative data from a second sample are used to establish the prevalence of PO injection among the subset of participants who reported having ever injected drugs and the frequency with which they injected POs and heroin. Quantitative data also establish how PO injection fits into participants’ broader opioid use trajectories and the associations between PO injection and key sociodemographic (e.g., socioeconomic status) and drug use-related (e.g., having ever overdosed) variables.

### Qualitative data collection and analysis

The qualitative portion of the analysis is based on data from 46 participants (ages 18–32) who were interviewed in the formative phase of the study. Participants lived in one of the five boroughs of NYC, reported having used POs for nonmedical reasons at least once in their lifetime (most reported use in the past 30 days), and spoke English or Spanish. Participants were referred by service providers (e.g., drug treatment programs, youth-focused harm reduction services), other research projects, or via chain-referral from other participants. Further details on the 46 participants included in the qualitative sample are reported elsewhere [19, 20].

Interviews were semi-structured, digitally audio-recorded, and lasted approximately 90 min. Interviews were transcribed verbatim, and the software program ATLAS.ti was used to facilitate the coding and analysis of qualitative data. Following a semantic thematic analysis approach [21, 22], the explicit meaning of participants’ words served as the basis for thematic coding. Interview transcripts were read to identify all quotes related to PO and heroin injection practices. Using these quotes, themes were identified on the basis of recurring patterns through multiple participants’ accounts. The themes established in this analysis were often related to the relatively more involved process of preparing and injecting an opioid pill (vs. injecting heroin). Themes corresponding to the general process of preparing a PO as opposed to heroin for injection included the “need for more water” and “applying heat.” Themes that were specific to injecting POs vs. heroin included “using larger syringes,” “multiple injections per injection episode (MIPIE),” and/or “reusing cottons.”

All names have been changed to pseudonyms to protect participants’ confidentiality, and some quotations have been slightly edited for clarity.

### Quantitative data collection

For the quantitative phase of the study, a different sample was recruited using respondent-driven sampling (RDS), a form of chain-referral sampling designed to engage hard-to-reach populations that uses participants’ personal network connections to drive recruitment. This method, which reaches people who may not frequent street settings, may yield a more representative sample than street recruitment. Using this method, an initial set of 20 “seeds” was directly recruited by research staff from referrals by harm reduction services, drug treatment programs, participants in the qualitative component, and other research projects. These participants completed structured assessments and were invited to refer up to three eligible peers from among their opioid-using contacts to participate in the study. This process was repeated with the seeds’ recruits and for successive sample waves leading to a total of 539 participants recruited from August 2014 to April 2016. Eligibility criteria included nonmedical use of POs and/or heroin use 3 or more times in the past 30 days, current residence in NYC, 18–29 years old, English-speaking, and the ability to provide informed consent. Participants were compensated $60.00 USD for completing the interview, and an additional incentive was provided for each eligible participant they referred. Further details on the RDS methods used in this study are reported in Mateu-Gelabert et al. [23].

Participants completed a computer-assisted, interviewer-assisted structured assessment lasting 90–120 min. The instrument included sociodemographic and behavioral questions (951 questions organized in 27 sections) related to the following: substance use and drug injection history and current practices, injection-related HIV/HCV risk behavior, opioid use and injection networks, and lifetime and recent overdose experiences, among other topics. The present analysis is based on the subset of the total sample who reported injecting any drug for nonmedical purposes at any point in their lifetime (*n =* 353/539, 66%). Because 6 participants did not respond to the questions specific to PO injection, analyses requiring these variables are based on a sample of 347.

### Variable definitions

In structured assessments and statistical analyses, nonmedical use of POs was defined as the “use of POs not prescribed for the respondent or use of these drugs only for the experience or feeling they caused” [24]. PO injection was defined as injecting any PO intended for oral intake. For the variables included in this report, “regular” injection was defined as injecting a given drug three or more times a week for at least 1 month. “Sporadic” injection was defined having injected a given drug at least once but not having done so regularly. Duration of regular injection of POs and heroin was measured by the number of months a participant had regularly injected the drug in their lifetime. The frequency of PO and heroin injection in the past 30 days was measured by the number of days each drug was injected. “Non-fatal overdose” was defined as any event in which a participant “lost consciousness, stopped breathing or was unresponsive as a result of drug use.” Opioid use trajectory variables included ages at first nonmedical PO use, first heroin use, first heroin injection, and first PO injection. Two injection risk variables were assessed, sharing syringes and sharing cookers, measured by the number of people with whom the sharing took place in the past 12 months. Sharing syringes was defined as receiving a syringe that had been previously used by someone else. Sharing cookers was defined as using a cooker someone else had previously used or using it simultaneously with someone else. All variables were based on self-report data except HCV antibody status, which was assessed with point-of-care rapid testing using the OraQuick Advance Rapid HCV Antibody Test (manufactured by OraSure Technologies, Inc., Bethlehem, PA).

### Quantitative analyses

All statistical analyses were conducted in the program R, versions 3.2.2 and 3.2.4 (R Core team, 2015) and IBM SPSS 25. For bivariable and multivariable analyses, response categories for lifetime PO injection were collapsed into two groups—never injected POs and ever injected POs—after a chi-squared test indicated a significant effect for several variables. First, binary associations of the three variables of interest (HCV-positive status, lifetime overdose, and lifetime PO injection) with a series of sociodemographic (gender, race/ethnicity, household income growing up, lifetime homelessness) and injection risk variables (syringe- and cooker-sharing in the past 12 months) were computed. Log ratios and *p* values were computed for all binary associations using a Wald chi-squared test, with a 95% confidence interval [25].. Following the strategy described in Hosmer et al., blocks of variables with *p* < 0.25 in bivariable analyses were then included in separate multivariable models for each of the three dependent variables of interest [26–28]. Multivariable models were run using generalized linear model (R version 3.2.4, glm_4.13-19), and adjusted odds ratios were computed using the model estimate. Results were verified with logistic regression in SPSS. Associations of PO injection with HCV-positive status and with non-fatal overdose were determined by separate multivariable models with HCV-positive status and non-fatal overdose as dependent variables, respectively. Potential sociodemographic predictors of lifetime PO injection were determined by a third multivariable model with PO injection as a dependent variable.

## Results

### Qualitative findings

Of the 46 qualitative interviewees, 27 were male, 18 female, and one transgender female. Participants were 25.3 years old on average (SD = 3.9 years; range = 18–32 years). In response to interview questions regarding participants’ opioid use, some participants provided detailed descriptions of their PO injection practices.

Participants described multiple methods for converting solid pills into injectable solutions. Each type of preparation method was tailored to the specific type and formulation of opioid. Participants preferred immediate-release (IR) oxycodone pills because they can be easily crushed and dissolved in water without the cumbersome process of trying to circumvent extended-release and/or abuse-deterrent technology. Respondents described the difficulties involved in trying to separate the opioid from the pill’s fillers and binders. Their stated goal was to maximize the amount of overall pill content that dissolved in water so the drug was suspended in a solution that was watery-enough to be drawn into a syringe. Bruce, a 26-year-old white male, described the method he used to prepare blue oxycodone pills for injection:

I’ll put the whole pill in there [in a cooker]. I’ll have a 1 cc syringe with at least 75 units of water in it, preferably warmish. I’ll squirt half on, I’ll take the back of a plunger of another needle…start crushing it as fine as I can, rest of the water mix it up with, again, the plunger, fold it, heat it for maybe five full seconds until your finger starts to hurt…I see the actual sediment, like the binders, you know it separates, you’ve got this pile of gook, and then you’ve got this blue water, it looks like a Smurf got melted. Throw in the cotton, and I suck it up, and then I’ll move the cotton around ‘cause a lot of it’s [the drug] stuck in the actual gel of the residue.

Bruce’s description is fairly representative of the techniques interviewees reported using to prepare opioid pills for injection. Converting extended-release and/or abuse-deterrent opioid formulations into an aqueous solution that is watery-enough to be drawn into a syringe typically requires a larger amount of water relative to the volume needed to dissolve the powder heroin available in NYC. The objective need for more water is of critical importance because the volume of the resulting pill-based solution is too large (> 0.5 cc) for the common syringe used by PWID in NYC (0.5 cc) to hold. To inject an opioid solution whose volume is greater than 0.5 cc requires a person to either administer multiple injections in a single injection episode using a 0.5-cc syringe [11, 12] or to use a larger syringe (≥ 1.0 cc) to inject the entire solution in a single injection.

Respondents also reported the need for heat when dissolving most opioid pill formulations, either heating the cooker with the flame from a lighter or heating the pill in a microwave or conventional oven. After adding water to the pill and adding heat, Bruce described the resulting material as viscous, referring to it as “gook,” and reported needing to perform additional steps to fully dissolve the pill. He described an intense and repeated process of manipulation whereby a water-soaked pill is repetitively crushed with the flat top of a syringe plunger while extra water is added and the filter is continuously repositioned in the cooker to create a solution that is watery enough to be effectively filtered and drawn into a syringe.

Some participants described reusing cottons that had been previously used by peers to filter PO-containing solutions in order to access the drug residue contained within them. In addition, participants also reported preparing an additional drug solution from the pill residue that remained in the cooker after the original solution was injected, despite being aware of the reduced opioid content associated with residue. For example, William, a 28-year-old, white male, stated:

I’ll do another shot out of it [the residue]. I’ll put water on it and I’ll mix it up again, and I’ll get a small rush out of it, not a lot but some… I’ll do my shot and then I’ll put water in it and I’ll mix it up and I’ll do it right after I do the shot. I’ll do two shots at once, yeah, or one right after the other.

As evident from William’s comment, and in contrast to injecting heroin, study participants typically described the injection of POs as more cumbersome and time-consuming. Dissolving heroin requires less water than extended-release and/or abuse-deterrent PO formulations and thus smaller-volume (e.g., 0.5 cc) syringes can be used to inject an entire heroin solution in a single injection. Because heroin in NYC is widely available in powder form and readily dissolves in water with little to no heat, needing less than 0.5 cc of water is standard practice. In the following excerpt, Elton, an 18-year-old male of Asian descent, summarizes why he prefers injecting heroin to pills: “As far as the pills go, I feel like it’s kind of inefficient. Maybe there is too much powder, doesn’t get absorbed fully, but for some reason, I don’t get the full rush as if you were shooting heroin.”

### Quantitative findings

The sociodemographic characteristics of the sub-sample of 353 PWID who participated in the study’s RDS phase are presented in Table 1. Most participants were male (65%), White/non-Latino (73%), and 36% grew up in households with annual incomes of less than $51,000). However, a considerable minority (23%) grew up in households with annual incomes of $101,000 or more. Thirty-nine percent reported never injecting POs, while 33% reported injecting POs sporadically, and 26% reported doing so regularly. In contrast, the vast majority (98%) had injected heroin and 89% had done so regularly.

**Table 1:** Characteristics of RDS-recruited Participants (*n* = 353)

Characteristic	Mean (SD)	***N*** (%)
Age	24 (3)	
Gender		
Male		230 (65)
Female		119 (34)
Transgender		4 (1)
Race/ethnicity		
White		259 (73)
Latino/a		66 (19)
Non-White and non-Latino/a		26 (7)
Household income growing up (annual)^1^		
$0–$50,000		126 (36)
$51,000–$100,000		116 (33)
$101,000 or more		81 (23)
Did not respond		30 (8)
Heroin and prescription opioid injection (lifetime)		
Never injected POs		139 (39)
Injected POs sporadically		116 (33)
Injected PO regularly^2^		92 (26)
Never injected heroin		5 (2)
Injected heroin sporadically		33 (9)
Injected heroin regularly^2^		314 (89)
Ever homeless		247 (70)
Ever overdosed		197 (56)
HCV antibody-positive		105 (30)

This table is based on the 353 participants who reported ever injecting drugs

^1^Thirty participants (9%) did not respond to the question about household income while growing up. For all other variables, less than 5% of data are missing

^2^“Regular” injection is defined as injecting 3 or more times per week for at least 1 month

Participants’ opioid use histories, drug used at first injection, and frequency of PO and heroin injection are presented in Table 2. On average, participants’ first nonmedical PO use occurred about 4 years prior to any opioid injection and 2.6 years prior to heroin use. Among those who reported ever injecting POs, first PO injection typically occurred shortly after the first heroin injection. Likewise, the drug used at first injection was overwhelmingly heroin (82%), followed by POs (7%).

**Table 2:** Prescription opioid (PO) and heroin injection history and behavior among young adult PWID (*n* = 353)

Variable	Mean (SD), median	***n*** (%)
Opioid use trajectory^1^		
Age at first nonmedical PO use	16.5 (3.0), 16	
Age at first heroin use	19.1 (3.5), 19	
Age at first heroin injection	20.4 (3.7), 20	
Age at first PO injection	20.6 (3.6), 20	
Drug used at first injection		
POs		26 (7)
Heroin		289 (82)
Crack		14 (4)
Other		24 (7)
Number of months injected POs regularly^2^ (lifetime)	10.2 (20.4), 0	
0 months		261 (74)
1–12 months		48 (14)
13–36 months		29 (8)
> 36 months		15 (4)
Number of months injected heroin regularly^2^ (lifetime)	34.5 (34.9), 24	
0 months		39 (11)
1–12 months		104 (29)
13–36 months		91 (26)
> 36 months		119 (34)
Number of days injected POs in the past 30 days	5.0 (16.0), 0	
0 days		304 (86)
1–7 days		32 (9)
8–15 days		14 (4)
> 15 days		3 (1)
Number of days injected heroin in the past 30 days	18.6 (11.6), 20	
0 days		56 (16)
1–7 days		54 (15)
8–15 days		44 (12)
> 15 days		199 (56)
Type of opioid injected in the past 30 days		
Heroin only		251 (71)
POs only		3 (1)
Heroin and POs		46 (13)
Neither heroin nor POs		53 (15)

This table is based on the 353 participants who reported ever injecting drugs

^1^Participants who did not inject POs/heroin were not included in the means and standard deviations

^2^“Regular” injection is defined as injecting 3 or more times per week for at least 1 month

On average, participants had regularly injected heroin for a total of 34.5 months (SD = 34.9), which is 24.3 months longer than they had regularly injected POs (10.2 months, SD = 20.4). A majority (84%) reported injecting heroin in the past month, with an average of 18.6 days of heroin injection in the past 30 days. Fewer participants (14%) had injected POs in the past month, averaging only 5 days of PO injection in the past 30 days (see Table 2).

Table 3 presents the bivariable and multivariable associations of lifetime PO injection with HCV-positive status when controlling for socio-demographics and injection risk variables. Lifetime homelessness, the number of people with whom the participant shared syringes in the past 12 months, and the number of people with whom the participant shared cookers in the past 12 months were associated with HCV-positive status in bivariable analyses and thus, along with household income growing up which had an omnibus *p* value < 0.25 (*p* = 0.18), were entered as covariates in determining the association between PO injection and HCV-positive status [27, 28]. PO injection was found to be associated with HCV-positive status (AOR 2.97, 1.50–5.86, *p* < 0.01).

**Table 3:** Correlates of HCV antibody-positive serostatus among young adult PWID (*n* = 347)

	HCV−	HCV+	Unadjusted OR (95% CI)	OR ***p*** value	AOR (95% CI)	AOR ***p*** value
*N* (%)	264 (71)	101 (29)	–	–	–	–
Injected POs (lifetime)						
No	117 (48)	22 (22)	Ref	Ref	Ref	Ref
Yes	129 (52)	79 (78)	3.26 (1.91–5.56)	< 0.01	2.28 (1.26–4.13)	< 0.01
Gender						
Male	89 (64)	138 (68)	Ref	Ref		
Female	50 (36)	66 (32)	1.01 (0.62–1.65)	0.96	–	–
Race/ethnicity						
Latino/a	46 (19)	18 (18)	Ref	Ref		
White	181 (74)	75 (74)	1.06 (0.58–1.94)	0.85	–	–
Non-Latino and non-White	17 (7)	8 (8)	1.20 (0.44–3.27)	0.13	–	–
Household income growing up (annual)						
$0–50 k	89 (38)	36 (43)	Ref	Ref		
$51–100 k	79 (34)	33 (39)	1.03 (0.59–1.81)	0.91		
> $100 k	65 (28)	15 (18)	0.57 (0.28–1.13)	0.11		
Homeless (lifetime)						
No	92 (37)	11 (11)	Ref	Ref	Ref	Ref
Yes	154 (63)	89 (89)	4.83 (2.46–9.52)	< 0.01	3.30 (1.62–6.71)	< 0.01
Number people shared syringes (past 12 months)						
0	155 (67)	45 (46)	Ref	Ref	Ref	Ref
1	59 (25)	28 (28)	1.64 (0.94–2.86)	0.08	1.16 (0.61–2.18)	0.65
2 or more	18 (8)	26 (26)	4.98 (2.50–9.88)	< 0.01	2.16 (0.96–4.87)	0.06
Number people shared cookers (past 12 months)						
0	108 (47)	24 (24)	Ref	Ref	Ref	Ref
1	53 (23)	17 (17)	1.44 (0.72–2.92)	0.31	1.38 (0.63–3.02)	0.41
2 or more	70 (30)	58 (59)	3.73 (2.12–6.55)	< 0.01	2.52 (1.31–4.83)	< 0.01

Sample total is 347 instead of 353 because 6 participants did not respond to the series of questions regarding PO injection

Table 4 presents a similar table to Table 3 except with the dependent variable being lifetime experience of non-fatal overdose. The same sociodemographic and injection risk variables associated with HCV-positive status were also found to be associated with non-fatal overdose in bivariable analyses: lifetime homelessness, the number of people with whom the participant shared syringes in the past 12 months, and the number of people with whom the participant shared cookers in the past 12 months. In addition, because the omnibus test for race/ethnicity and for household income growing up had *p* values of < 0.25 (*p* = 0.17 and *p* = 0.18, respectively), these variables were also included in the model. PO injection was found to be associated with non-fatal overdose (AOR 2.51, 1.47–4.28, *p* < 0.01) when controlling for these five sociodemographic and injection risk variables.

**Table 4:** Correlates of non-fatal overdose among young adult PWID (*n* = 340)

	Never overdosed	Overdosed at least once	Unadjusted OR (95% CI)	OR ***p*** value	AOR (95% CI)	AOR ***p*** value
*N* (%)	146 (43)	194 (57)	–	–	–	–
Injected POs (lifetime)						
No	80 (55)	57 (29)	Ref	Ref	Ref	Ref
Yes	66 (45)	137 (71)	2.91 (1.86–4.56)	< 0.01	2.51 (1.53–4.11)	< 0.01
Gender						
Male	97 (67)	124 (65)	Ref	Ref		
Female	47 (33)	68 (35)	1.13 (0.72–1.79)	0.60	–	–
Race/ethnicity						
Latino/a	33 (23)	29 (15)	Ref	Ref		
White	101 (70)	151 (78)	1.70 (0.97–2.98)	0.06	–	–
Non-Latino/non-White	11 (8)	13 (7)	1.34 (0.52–3.46)	0.54	–	–
Household income growing up (annual)						
$0–50 k	61 (45)	63 (36)	Ref	Ref		
$51–100 k	48 (35)	60 (35)	1.21 (0.72–2.03)	0.47		
> $100 k	28 (20)	50 (29)	1.73 (0.97–3.09)	0.06		
Homeless (lifetime)						
No	53 (36)	47 (24)	Ref	Ref	Ref	Ref
Yes	93 (64)	146 (76)	1.77 (1.10–2.84)	0.02	1.10 (0.65–1.85)	0.73
Number people shared syringes (past 12 months)						
0	96 (70)	98 (52)	Ref	Ref	Ref	Ref
1	33 (24)	54 (29)	1.60 (0.96–2.69)	0.07	1.21 (0.68–2.15)	0.51
2 or more	8 (6)	35 (19)	4.29 (1.89–9.71)	< 0.01	2.25 (0.87–5.82)	0.09
Number people shared cookers (past 12 months)						
0	69 (51)	57 (30)	Ref	Ref	Ref	Ref
1	33 (24)	37 (20)	1.36 (0.76–2.44)	0.31	1.34 (0.71–2.55)	0.37
2 or more	34 (25)	93 (50)	3.31 (1.96–5.61)	< 0.01	2.50 (1.39–4.50)	< 0.01

Sample total is 340 instead of 353 because 6 participants did not respond to the questions regarding PO injection and 7 additional participants did not respond to the questions regarding overdose

Table 5 presents the bivariable and multivariable associations of lifetime PO injection with the same series of sociodemographic independent variables. Gender and race/ethnicity were not significantly associated with PO injection in bivariable analyses. Nevertheless, race/ethnicity was included in the multivariable analysis due to its omnibus *p* value of < 0.25 (*p* = 0.10). In multivariable logistic regression, compared to growing up in low-income households, PO injection was significantly correlated with growing up in middle- (AOR 1.86, 1.07–3.21, *p* = 0.03), or high-income households (AOR 2.54, 1.35–4.76, *p* < 0.01). PO injection was also associated with lifetime homelessness (AOR 2.93, 1.75–4.91, *p* < 0.01).

**Table 5:** Correlation between sociodemographics and lifetime PO injection among young adult PWID (*n* = 347)

	Never injected POs	Ever injected POs	Unadjusted OR (95% CI)	OR ***p*** value	AOR (95% CI)	AOR ***p*** value
*N* (%)	139 (40)	208 (60)	–	–	–	–
Gender						
Male	89 (64)	138 (68)	Ref	Ref		
Female	50 (36)	66 (32)	0.85 (0.54–1.34)	0.49	–	–
Race/ethnicity						
Latino/a	27 (19)	37 (18)	Ref	Ref		
White	97 (70)	159 (77)	1.20 (0.69–2.09)	0.53	–	–
Non-Latino and non-White	15 (11)	10 (5)	0.49 (0.19–1.25)	0.13	–	–
Household income growing up (annual)						
$0–50 k	62 (48)	63 (34)	Ref	Ref	Ref	Ref
$51–100 k	43 (33)	69 (37)	1.58 (0.94–2.65)	0.08	1.89 (1.10–3.26)	0.02
> $100 k	25 (19)	55 (29)	2.17 (1.20–3.90)	0.01	2.74 (1.45–5.06)	< 0.01
Homeless (lifetime)						
No	56 (40)	47 (23)	Ref	Ref	Ref	Ref
Yes	83 (60)	160 (77)	2.30 (1.44–3.67)	< 0.01	2.85 (1.71–4.74)	< 0.01

Sample total is 347 instead of 353 because 6 participants did not respond to the questions regarding PO injection

## Discussion

This paper provides a qualitative description of the PO injection practices of young PWID in NYC. Qualitative interview findings reveal that PO injection often necessitates the use of 1.0 cc syringes or the administration of multiple injections with smaller syringes (0.5 cc) due to the additional water that is needed to dissolve extended-release and/or abuse-deterrent opioid pills in aqueous solution. These multi-step pill injection practices can increase the likelihood of sharing and cross-contaminating injection equipment, thereby increasing the risk of HIV [29] and HCV transmission [6, 28, 30]. Our findings corroborate results from other qualitative studies that describe the unique mechanics involved in preparing and injecting POs [8, 11]. By contrast, the mechanics required to prepare and inject heroin—particularly, the powder form of heroin that predominates in the Eastern part of the USA—are considerably less cumbersome, and consequently, intrinsically less risky. The nominal amount of residue that remains in cookers after episodes of heroin injection reduces the likelihood that PWID will be motivated to consume or share “rinse shots,” as was reported by study’s participants in reference to injecting POs, as well as by Bruneau et al. and Broz et al. [7, 11].

Quantitative results indicate that, in addition to injecting heroin, a majority of young PWID in our sample (59%) have also injected POs. The prevalence of PO injection, however, is considerably lower than the 75% reported by Bruneau et al. in Montreal and the 89% reported by Havens et al. in rural Appalachia [3, 7]. The lower prevalence of PO injection in our NYC sample might be explained by regional differences in drug markets. Heroin in NYC is widely available and significantly cheaper than diverted POs, allowing PWID to choose between POs and heroin based on their income-generating strategies and SES, whereas in Canada [29], heroin is available but more expensive than POs, and in certain rural areas of the USA, like Kentucky [3], heroin is difficult to access, which can necessitate more frequent PO injection, even in places where POs are exceedingly expensive (e.g., Scott County, Indiana).

Our qualitative findings illustrate how the preparation and injection practices associated with the parenteral administration of POs (e.g., multiple drug washes, reuse of drug paraphernalia containing drug residue, multiple injections per injection episode) place PWID at increased risk for blood-borne infection. Adjusted odds ratios from multivariable analysis indicate that participants who tested HCV antibody-positive had 2.3 times the odds of having injected POs than those who had never injected them. These results support similar findings reported in both urban [4, 7] and non-urban settings [3, 6]. Taken together, our research findings from NYC contribute to an emerging body of literature indicating that PO injection is independently associated with increased risk of HCV infection. This heightened risk may explain, in part, the sharp increase in acute HCV infections in many jurisdictions throughout the USA where PO injection is prevalent [31]. It also raises the possibility of an HIV outbreak driven by sharing injection paraphernalia other than syringes when PWID reinsert their own used needle into the cooker in preparation of multiple washes [32]. Additionally, to the extent that PO injection motivates PWID to re-use drug-residue-containing filters or cottons, it could potentially heighten risk for endocarditis, as damp filters provide an ideal breeding ground for bacteria.

In this paper, we also contrast patterns of heroin and PO injection among young adult PWID in NYC. In a drug market with a steady heroin supply, such as NYC, the preponderance of young PWID in this study initiated their opioid use with oral intake of POs at about 16 years old. Oral consumption of POs occurred for an average of 2–3 years prior to the first heroin use at age 19. Despite years of nonmedical PO use without heroin, a large majority of PWID (82%) chose heroin as their first injected drug. In sharp contrast to the reported 62% of PWID in rural Appalachia who initiated injection with POs [3], only 7% of young PWID in our sample chose POs as their first injected drug. This geographic variation may indicate that in areas where both heroin and POs are readily accessible, opioid users are far more likely to choose heroin instead of POs as the first drug they inject. This might also result from wanting to avoid the complicated hassle of preparing PO for injection.

Despite the predominance of heroin as the drug of choice for initiating injection, a majority of participants (59%) did report ever having injected POs, indicating a significant prevalence of PO injection even in an urban location with a well-established heroin market. The widespread availability of heroin in NYC, however, appears to influence the frequency with which participants reported injecting POs relative to heroin. Although many participants had experimented with injecting POs, it was not often a regular practice: only 26% of respondents reported regular PO injection for at least 1 month (vs. 89% who reported regular heroin injection). On average, these young PWID, who ranged in age from 18 to 29 years old, reported having injected heroin regularly for 2.8 years—about 2 years longer than their reported average duration of regular PO injection (0.8 years). Similarly, when considering the number of days participants reported injecting POs vs. heroin in the past 30 days, heroin was injected far more frequently: 84% reported having injected heroin in the past 30 days, for an average of 18.6 days, while only 14% report having injected POs in the past 30 days, for an average of only 5 days. Nevertheless, the high prevalence of occasional PO injection still poses significant health risks for PWID.

Analyses presented here also indicate that PO injection is significantly correlated with lifetime experience of non-fatal overdose. Similar to other studies [14, 15], our bivariable results indicate a strong correlation between PO injection and overdose. In contrast to these previous studies, however, the association of overdose with PO injection in the current study remained significant in the multivariable model, suggesting a robust association in this sample of young New Yorkers who inject drugs. There are several possible explanations for the observed relationship between PO injection and overdose. On the one hand, PO injectors in this sample are more likely to have grown up in middle- or upper-income families; ergo, they may have more money to spend on opioids and other drugs, which may allow for higher drug intake or polysubstance use, thereby contributing to increased overdose risk. PO injection could also be serving as a marker for a subset of youth who engage in a range of high-risk drug-use behaviors, including behaviors, such as binging on drugs and/or engaging in polysubstance use, that are known to increase risk of overdose.

PO injection was also correlated with having been raised in a higher-income household. This association may possibly reflect the ability of people with higher incomes to continue purchasing POs while concurrently using heroin, which tends to occur in drug contexts where heroin is cheaper and easier to access than POs [20]. Yet, to complicate matters, findings also indicate that lifetime homelessness is significantly correlated with PO injection. This may show that different subsets of the population of young PWID in NYC may be engaging in the injection of multiple drugs [4].

This study has some limitations, particularly pertaining to the use of self-reported and cross-sectional data. Participants’ ability to recall past events and behaviors, including those that may have occurred years prior, is unknown. There is also the possibility that self-reported data is susceptible to social desirability bias, especially given the sensitive and stigmatized topic of illicit drug use. Because of the cross-sectional nature of the data, our findings cannot establish causation, only correlation. Additionally, as this sample was comprised entirely of young adults who live in NYC, results may not generalize to other populations, especially those in non-urban areas. The use of a non-random recruitment strategy—respondent-driven sampling—may also have introduced bias into the sample that limits the generalizability of the findings. An additional limitation in the qualitative data is participants’ imprecise description of the pills used for injection, thus limiting our ability to determine which preparation methods are most common to specific PO formulations (regular extended-release and/or abuse-deterrent).

Study results illustrate the need for harm reduction strategies to address the specific health risks, particularly with regard to HCV transmission, posed by the injection of POs. Knowledgeable people who use drugs, prevention projects and agencies should explicitly inform PWID of the increased viral transmission risk associated with PO injection practices and how to mitigate such risk, mainly by always using new, sterile equipment for every injection, and avoiding the sharing of any injection paraphernalia (syringes, cookers, filters, diluting water, and water containers), even if it contains drug residue. In group injection situations where people are doing multiple injections per injection episode, it can be very difficult to avoid accidental cross-contamination. If there is any sharing or splitting of drug solution from a communal cooker, then all PWID involved need to use a sterile syringe for each injection. In situations where individuals will be re-using their own syringes for repeated injections, all those involved in the group injection should mix their own drugs in their own cooker. If larger syringes are to be used, those with detachable needles should never be used, as they hold a larger amount of residual blood and therefore increase the risk of transmitting HCV or HIV [33]. If sharing POs and injecting, users should try to split the pill before breaking it down for injection, with each individual using exclusively their own injection equipment. Recent research suggests that heating a PO-containing drug solution until boiling reduces the risk of HIV transmission [32]; the extent to which this procedure may protect against HCV transmission is yet unknown but warrants future research.

In summary, these results suggest that a high proportion of young PWID in NYC have injected POs, although it appears to be less frequent than heroin injection. Further, lifetime experience of non-fatal overdose and HCV antibody-positive serostatus were independently associated with having ever injected POs. Existing harm reduction efforts should inform PWID of the increased risks associated with injecting POs and tailor harm reduction messages to address the risky practices associated with preparing and injecting POs, including multiple injections per injection episode and the re-use of drug residue-containing cookers and filters.

## Data Availability

All data generated or analyzed during this study are unavailable due to the presence of identifiers.

## Abbreviations

PO: Prescription opioid
PWID: People who inject drugs
NYC: New York City
HIV: Human immunodeficiency virus
HCV: Hepatitis C virus
RDS: Respondent-driven sampling
IR: Immediate release
